# Defect-engineered TiO_2_ Hollow Spiny Nanocubes for Phenol Degradation under Visible Light Irradiation

**DOI:** 10.1038/s41598-018-24353-8

**Published:** 2018-04-12

**Authors:** Xiaolan Kang, Xue-Zhi Song, Ying Han, Junkai Cao, Zhenquan Tan

**Affiliations:** School of Petroleum and Chemical Engineering, Dalian University of Technology, Panjin, 124221 P. R. China

## Abstract

Herein, we mainly report a strategy for the facile synthesis of defect-engineered F-doped well-defined TiO2 hollow spiny nanocubes, constructed from NH_4_TiOF_3_ as precursor. The topological transformation of NH_4_TiOF_3_ mesocrystal is accompanied with fluorine anion releasing, which can be used as doping source to synthesize F-doped TiO_2_. Our result shows that the introduction of oxygen vacancies (Vo’s) and F dopant can be further achieved by a moderate photoreduction process. The as prepared sample is beneficial to improve photocatalystic degradation and Photoelectrochemical (PEC) efficiency under visible light irradiation. And this improvement in photocatalytic and photoelectrocatalytic performance can be ascribed to the significant enhancement of visible light absorption and separation of excited charges resulted from the presence of oxygen vacancies, F^−^ ions and hollow structure of TiO_2_.

## Introduction

Photocatalysis is a prospective strategy for the degradation of pollutants, and mass hydrogen production from water^1,2^. As one of the most important photocatalyst, TiO_2_ has drawn much attention because of its excellent chemical stability, a suitable band structure and low cost. These merits contribute the possibility of various photocatalytic reactions to continue under ultraviolet (UV) illumination^3^. Many endeavors have been dedicated to enhance the photoactivity of TiO_2_ based nanomaterials, such as defect engineering^4,5^, crystal facets regulation, the surface/interface control^6,7^, and the complex structures^8^. Chen *et al*.^9^ have first reported the preparation of black TiO_2_ nanomaterials by treating TiO_2_ under a 20.0-bar pure H_2_ atmosphere at 200 °C for about 5 days. The as-prepared black TiO_2_ nanomaterial showed a disordered surface layer, exhibiting enhanced solar light absorption efficiency and photocatalytic activity. TiO_2_ with well-controlled defects such as oxygen vacancy and trivalent titanium, which can introduce mid-gap states, will lead up to a substantially enhanced optical absorption in the visible light and even near-infrared light region. Besides, this disorder can also facilitate the separation and migration of photogenerated carriers, effectively inhibit the fast recombination of electrons and holes to improve the photoactivity^10,11^. However, due to the excellent stability, rigorous reaction conditions are inevitable to induce different kinds of defects into TiO_2_, for example, electron beams irradiation, high temperatures or pressures and the long hydrogenation time^4,12^. It is an urgent need to seek convenient, economical and secure approach to synthesize defect-engineered TiO_2_.

F-doping is a widely used route to improve the photocatalytic efficiency. Xu *et al*.^13^ revealed that oxygen vacancies were created by F dopant, and the enhanced photocatalytic efficiency of F-TiO_2_ film is due to the extrinsic absorption through these oxygen vacancies instead of the excitation of the bulk TiO_2_ intrinsic absorption band. The enhanced photocatalytic activity of F-TiO_2_ nanostructure in the photodegradation of formic acid under visible light irradiation is reported by Dozzi *et al*.^14^ to the reduced chance for charge carrier recombination. As an anionic dopant, F^−^ doping, which replace O^2−^ ions in TiO_2_ lattice, facilitates the generation of oxygen vacancies and Ti^3+^ centers originated from the charge compensation^15,16^. Besides, F-doped TiO_2_ is also reported with high surface wettability^17^. F^−^ ions, with highly electronegative, physically adsorbed on TiO_2_ surface can promote the photo-generated holes migrate from bulk TiO_2_ to its surface^18^, which suppresses the photo-induced charges recombination in TiO_2_.

Due to the covalent interaction between Ti and F, NH_4_TiOF_3_ was served as fluorine ions dopant source to prepare a homogeneous F^−^doped TiO_2_^19,20^. NH_4_TiOF_3_ is a mesocrystal and its structure is very similar with anatase TiO_2_^21^. Previous studies have shown that washing with aqueous H_3_BO_3_ or pyrolysis can promote the anisotropic dissolution of NH_4_TiOF_3_ and cause the crystal topological transformation from NH_4_TiOF_3_ to TiO_2_, accompanying with N and F releasing^22,23^. The released F provides the possibility for doping. Furthermore, during the process of topotactic transformation, TiO_2_ with ordered and hollow spiny structures can be constructed.

Hierarchical hollow structured TiO_2_ nanomaterials have attracted extensive attention. The appropriate inner cavity allows for multiple reflection of light within its interior voids, greatly enhancing the absorption of the light source^24^. Also, the unique hollow structure benefits the increase of specific surface area and reduces the transport lengths for charge carriers, accelerating the migration and separation of photogenerated carriers^25^. However, traditional methods to synthesize TiO_2_ hollow nanostructures relied on energy-consuming and complicated hard or soft templates methods^26,27^. As an alternative, exploring template-free approaches based on different mechanisms become necessary.

Herein, defect-engineered F-doped TiO_2_ hollow spiny nanocubes was successfully constructed with NH_4_TiOF_3_ as precursor. Topological transformation of NH_4_TiOF_3_ mesocrystal is accompanied by the release of F^−^, which can be used as doping source to synthesize doped TiO_2_. The results show that the introduction of oxygen vacancies (Vo’s) and F dopant can be further achieved by a moderate photoreduction process. The presence of oxygen vacancies were reported to induce the formation of sub-bands below CB, which enabled the entrapment of visible light photons. The synthesized TiO_2_ crystals exhibit excellent catalytic activity and stability in photocatalytic degradation and PEC measurement under visible light.

## Results Section

**Figure 1:**
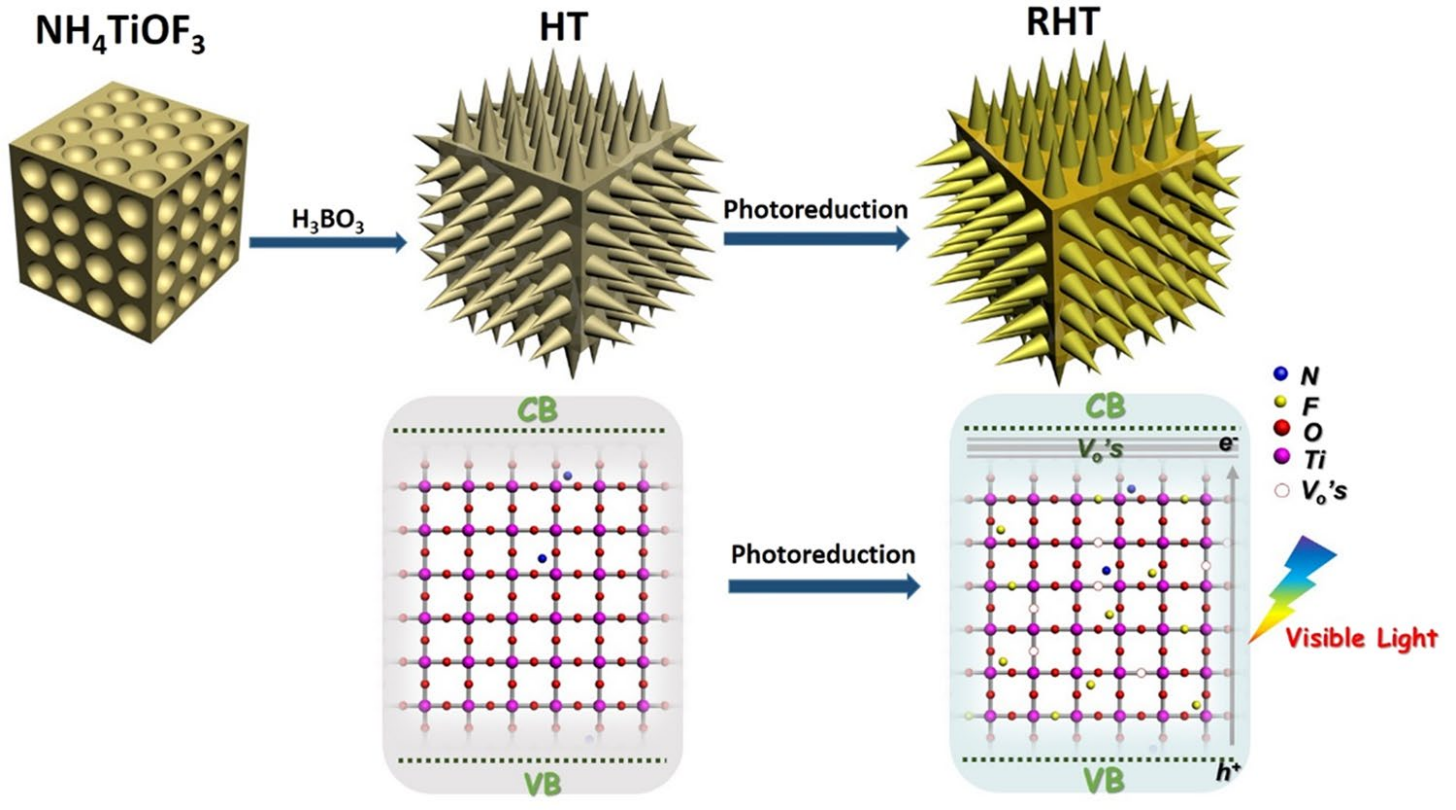
Schematic of the synthesis of RHT, and the corresponding Energy band diagrams.

As illustrated in Fig. 1, the non-porous mesocrystal NH_4_TiOF_3_ nanocubes with highly oriented nanoparticles were fabricated via a hydrothermal reaction. Then, after treatment with H_3_BO_3_, the intermediate NH_4_TiOF_3_ nanocubes began to topotactically transform to hollow spiny anatase TiO_2_ with a dominant {001} facet. After subsequent photoassisted treating with the irradiation of Xenon-lamp, oxygen vacancies as well as F^−^ ions were induced into TiO_2_, simultaneously.

**Figure 2:**
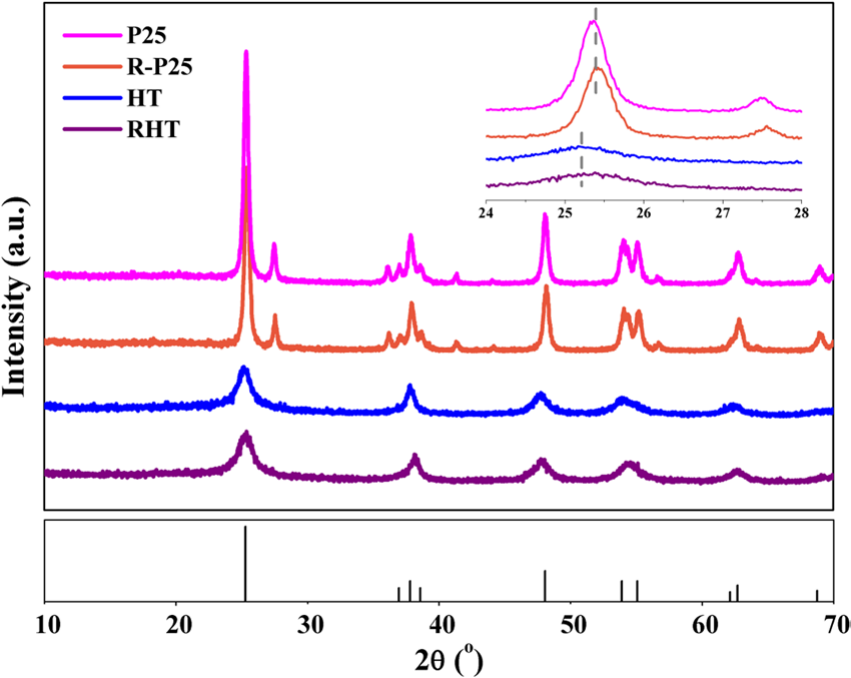
XRD patterns of the as-prepared samples.

The X-ray diffraction (XRD) analysis of the prepared NH_4_TiOF_3_ nanocubes possess a set of diffraction peaks and can be well indexed to crystalline NH_4_TiOF_3_ (JCPDS NO.54–0239, Figure S1). After boric acid treatment, the intermediate NH_4_TiOF_3_ was transformed to anatase TiO_2_, which is characterized by the XRD patterns (JCPDS No.21–1272) in Fig. 2. The crystal composition and phase of unreduced TiO_2_ (HT) sample were persevered with prolonged treatment time. To be notable, in comparison with the unreduced samples, the enlarged diffraction peaks of {101} facet of photoreduced TiO_2_ (RHT) and R-P25 moved toward the higher angle range, indicating the shrink of the crystal lattice^28^. This shrink is probably associated with the exist of oxygen vacancies after photoreduction treatment.

**Figure 3:**
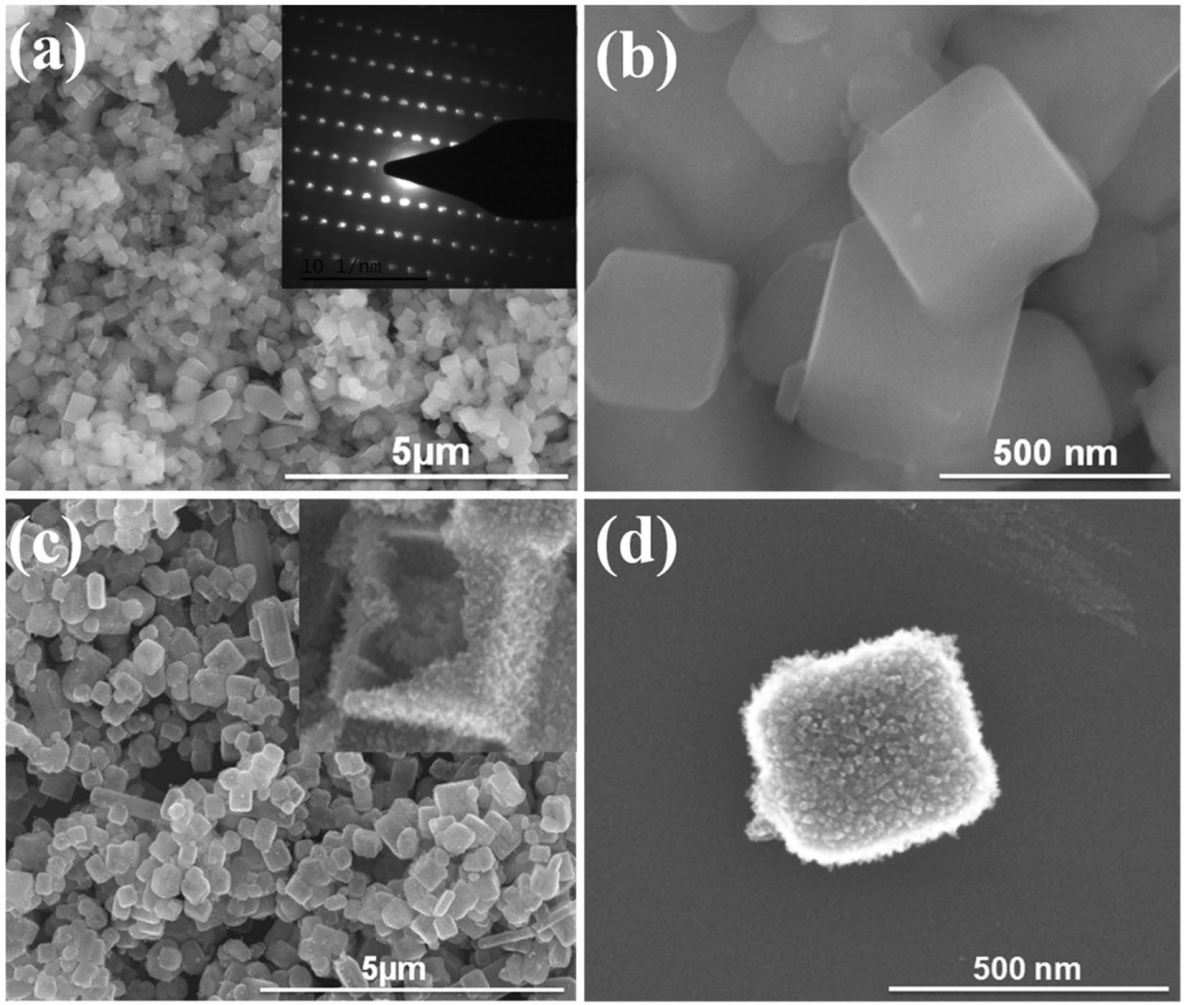
SEM images of the as prepared samples: (**a**,**b**) NH_4_TiOF_3_, (**c**,**d**) HT.

A panoramic Scanning Electron Microscopy (SEM) images (Fig. 3) as well as the transmission electron microscopy (TEM) images (Figure S2a) of the as-prepared NH_4_TiOF_3_ sample clearly demonstrate that well-defined uniform NH_4_TiOF_3_ nanocubes with the size of 200–400 nm were successful synthesized by the facile hydrothermal method. Single-crystal diffraction pattern of NH_4_TiOF_3_ was recorded by a selected-area electron diffraction (SAED), the symmetric pattern indicates the ordered alignment of a great number of nanocrystals (inset in Figure S2b). The same result was also acquired from the images of high resolution transmission electron microscopy (HRTEM) in Figure S2b. The obtained HT cubic sample is rough with the original particle size, and an enlarged SEM image in Fig. 3c suggests the hollow structure of HT, constructed by nanothorny primary building particles.

**Figure 4:**
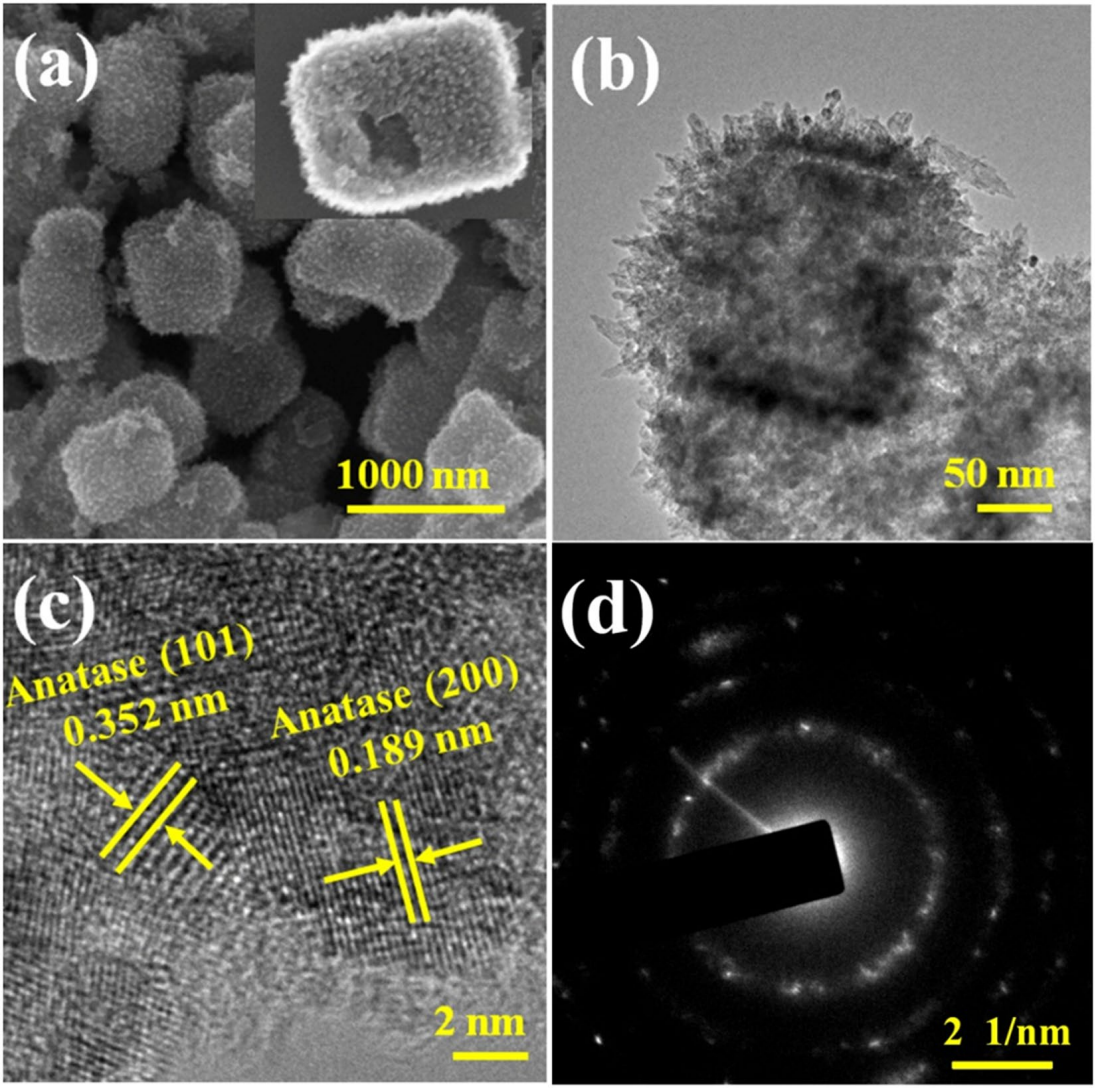
(**a**) TEM, (**b**) SEM, (**c**) HRTEM and (**d**) SAED images of the as-prepared RHT.

The same hierarchical hollow structure of RHT hollow nanocubes (Fig. 4a–d) obtained in the present work indicates that photoassisted process would not change morphology of the sample. However, the hollow structure could be destroyed by ultrasound and grind (Figure S3). Interestingly, according to the TEM images in Fig. 4b, RHT sample features a hollow structure consisting of nano-thorns, arranging in order with the same stretching direction. Furthermore, from the images of HRTEM (Fig. 4c), these nanothorns are well-defined thin sheets with the lattice spacing of 0.189 nm, in good agreement well with the (200) and (020) lattice planes of anatase type TiO_2_, which can be indexed into the high energy {001} facets. SAED image, corresponding to the TiO_2_ nanocubes also confirms this particular structure in Fig. 4d. These different kinds of diffraction rings in SAED pattern illustrate that nanothorns are constituted with multiple crystal facets, which suggests its multi-crystalline structure.

**Figure 5:**
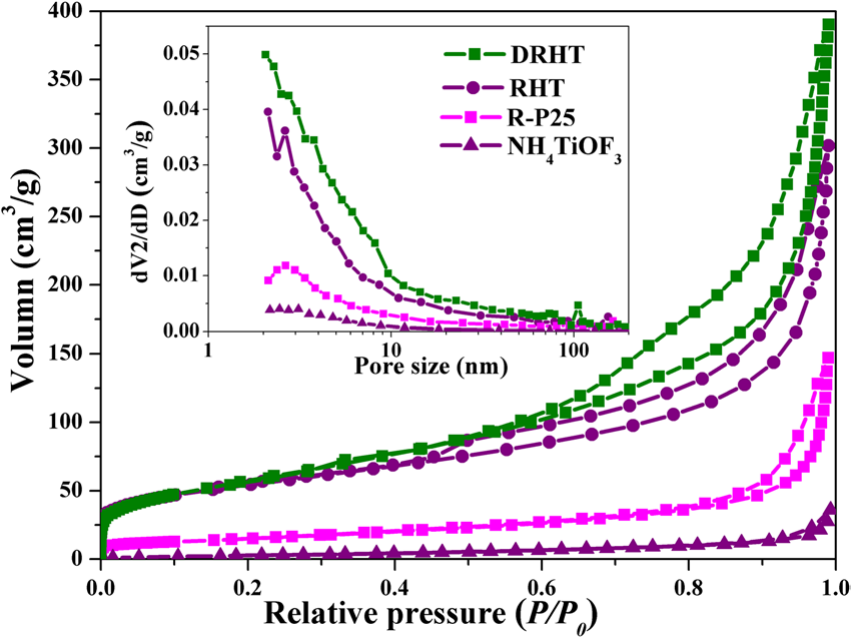
N_2_ adsorption–desorption isotherms and pore size distribution (inset) of Degussa P25 and as-synthesized samples.

During the topotactic growth and photoreduction process, structural analysis was performed using N_2_ adsorption-desorption isotherm (Fig. 5) with its corresponding pore size distribution (inset in Fig. 5). Both RHT and DRHT samples exhibit type-IV isotherms with hysteresis loop, indicating their mesoporous feature. The Brunauer-Emmett-Teller specific surface area (BET) of the NH_4_TiOF_3_ precursor (12.7 m^2^/g) was significantly increased to 191.9 m^2^/g of RHT after H_3_BO_3_ treatment, reasonably originating from its unique hollow structure decorated with nanothorns. Moreover, the large specific surface area would facilitate the contact between ethanol reagent and the as prepared samples during the photoreduction process. More information can be seen in Table S1.

**Figure 6:**
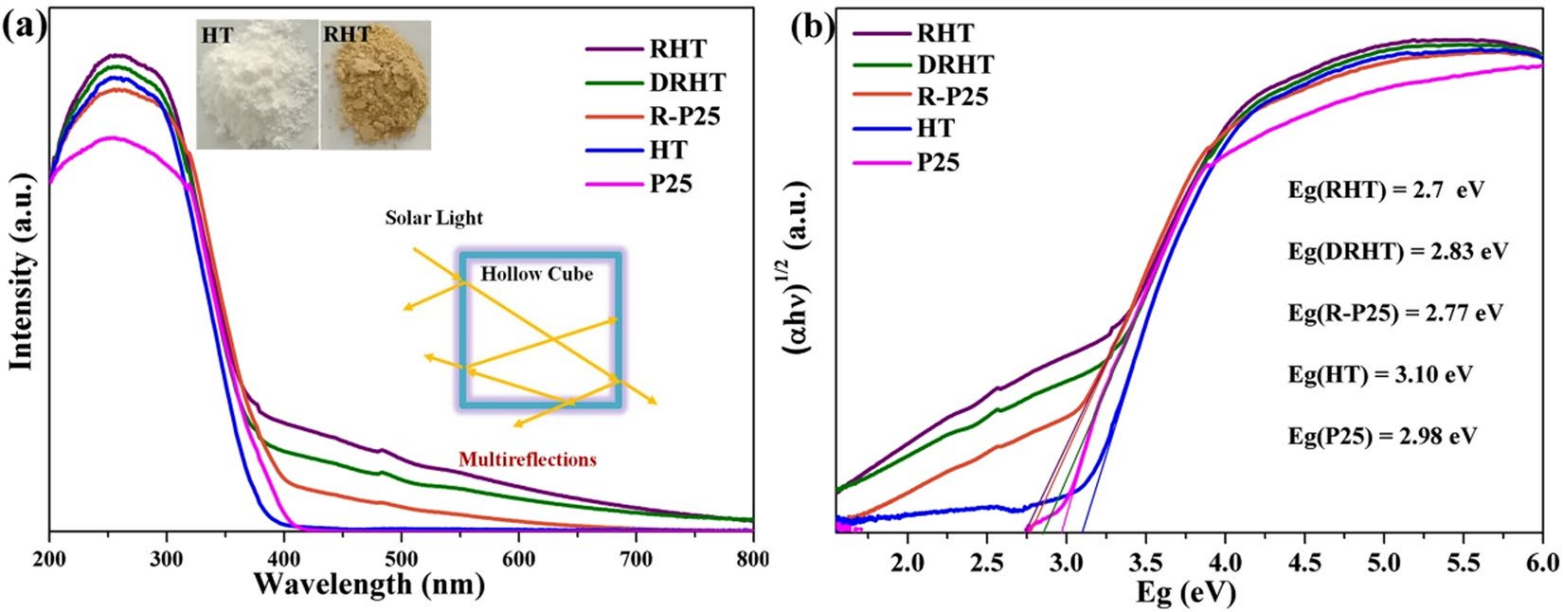
(**a**) The UV–vis diffuses reflectance spectra and (**b**) Kübelka Münk plots of the as-prepared samples.

The light absorption performances of as prepared samples were detected using a UV-vis diffuse reflectance spectra (DRS) (Fig. 6a). These reduced samples, RHT, DRHT and R-P25, exhibit relatively higher visible light absorption, as opposed to the pristine samples of P25 and HT. This extended absorbance can be correlated to the trend of change from white color to earthy yellow, representing the existence of oxygen vacancies in reduced TiO_2_. Therefore, surface modification was achieved by a facile and mild photoassisted treatment of TiO_2_ nanoparticles, which is reflected in improved visible light absorption as well as the color change. Similarly, the bandgap values of the as prepared samples (Fig. 6b), estimated from the edge of the Tauc plot, were narrowed from 3.1 to 2.7 eV. These spectral changes suggest that defect states were formed below the conduction band edge after the photoreduction treatment, since little change was shown in the valence band (Figure S4). Furthermore, RHT shows the stronger light absorption than the crushed DRHT sample, which attributes to the multiple reflection of light within the nanocube voids.

**Figure 7:**
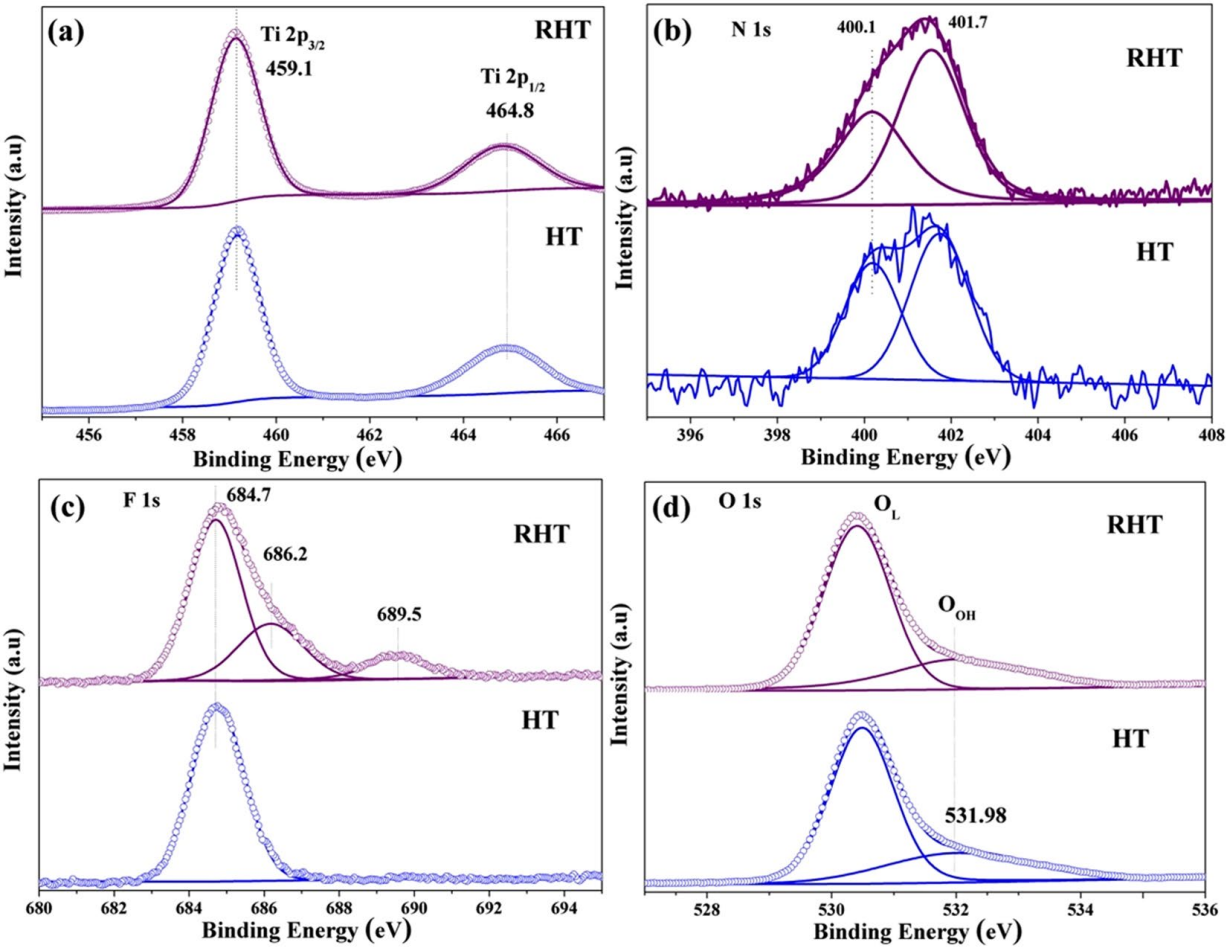
XPS spectra of (**a**) Ti 2p, (**b**) N 1s, (**c**) F 1s and (**d**) O 1s for HT and RHT samples.

The change of element binding energy and chemical bonding on samples’ surface were evaluated with X-ray photoelectron spectroscopy (XPS)^29^. The binding energies of Ti 2p_3/2_ and Ti 2p_1/2_ which can be detected at ~458.6 eV and ~464.5 eV, respectively, are typical for Ti^4+^ species in TiO_2_^12,28^. Additionally, the almost identical Ti 2p spectra of RHT and HT and the absence of any Ti^3+^ signals^30^ suggests that no titanium with lower oxidation states were observed on the surface of RHT samples (Fig. 7a). This phenomenon can be explained by the readily oxidized of Ti^3+^ by O_2_ in air or dissolved oxygen in water. The same result can also be seen in P25 and R-P25 (Figure S5). Figure 7b displays the N 1s XPS spectra, N 1s binding energy which is observed at ~401.7 eV and ~400.1 eV, can be positively ascribed to interstitial nitrogen species. The former N component refers to some forms of chemisorbed nitrogen, whereas the latter one is attributed to oxidized N in the form of Ti-O-N linkages, both of these have some important role in the photocatalytic activity of TiO2^31,32^.

F 1s XPS spectra of TiO_2_ nanocubes before and after photoreduction treatment are displayed in Fig. 7c. The lower binding energy of ~684.7 eV in both HT and RHT samples can be recognized to F^−^ ions physically adsorbed on TiO_2_ nanostructure’s surface^33^. However, it is clearly observed that two extra peaks were introduced by photoreduced treatment: F atoms replaces the O atoms in TiO_2_ crystal lattice, forming F-Ti bonds, can be demonstrated by the higher binding energy in 689.5 eV; while another one located at 686.2 eV was ascribed to the F^−^ in interstitial sites of solid TiO_2_, (interstitial doping)^15,34^. Therefore, it is reasonable to assume that photoreduction process can promote F^−^ incorporation into TiO_2_ lattice, forming F^−^ doped TiO_2_. STEM-EDS image of the RHT sample (Figure S6) clearly shows that N, F were uniformly dispersed on the surface of TiO_2_, in consistent with the XPS results.

Two different peaks were acquired by deconvoluting the O 1s peaks (Fig. 7d and Figure S5). The higher binding energy represents surface Ti-OH groups (O_OH_) and the lower one is surface lattice oxygen species, that is Ti-O-Ti (O_L_)^3,8^. As compared with P25, photoreduced R-P25 sample exhibits enhanced intensity of the O_OH_ peaks, indicating the increased O_OH_ concentration by photoreduction treatment. Interestingly, an opposite result was obtained between RHT and HT samples, the O_OH_ concentration of RHT decreases a little as compared with HT. This decrease can be explained by F doping. During this doping process, reactions between Ti-OH and F^−^ would lead to the formation of Ti-F bond, which definitely decreasing the surface O_OH_. Specific results are provided by Table S2.

**Figure 8:**
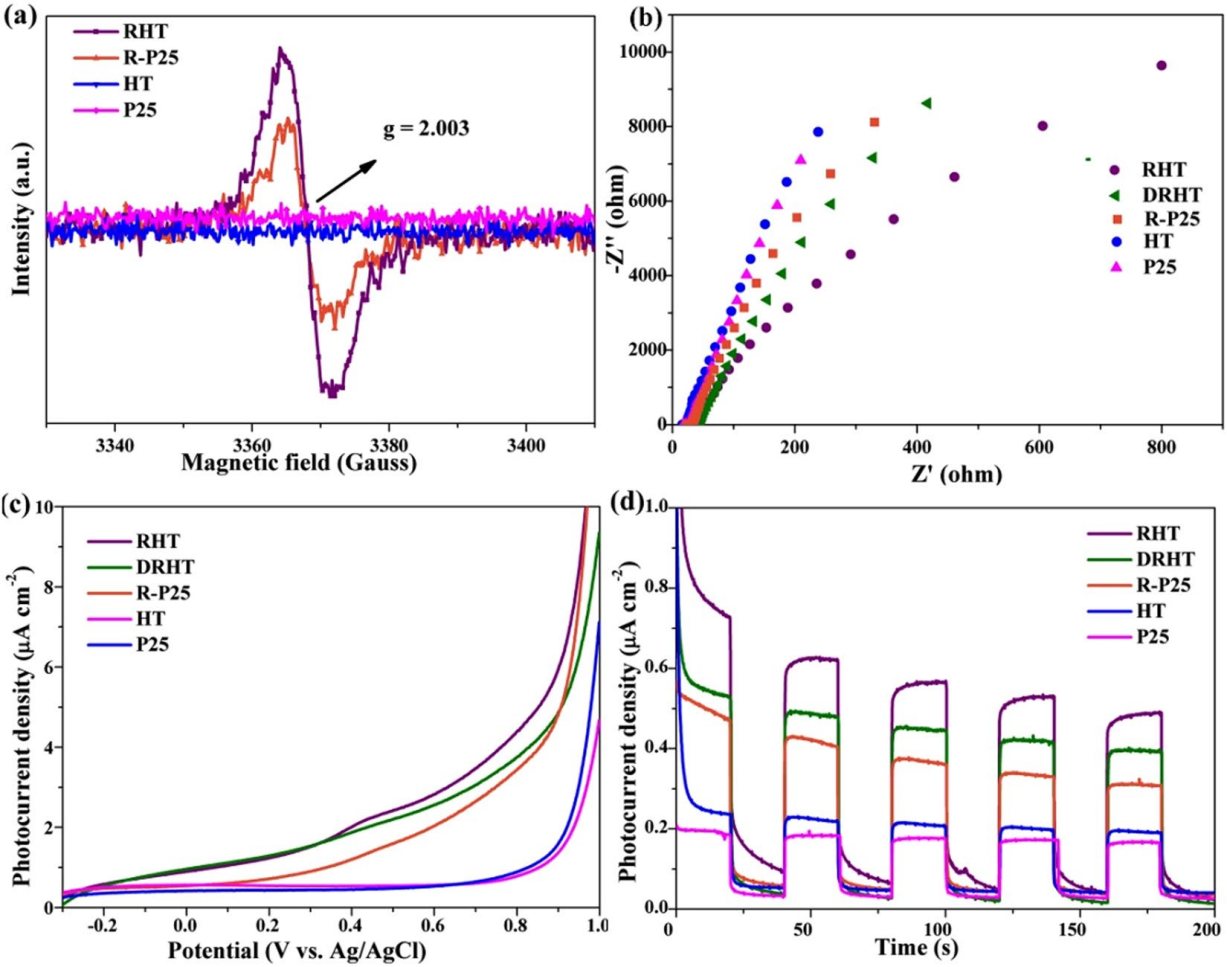
(**a**) ESR spectra at room temperature, (**b**) The Nyquist plots of electrochemical impedance, (**c**) Linear sweep voltammograms and (**d**) Chronoamperometry tests and under visible light irradiation of the as-prepared samples.

Electron spin resonance (ESR) spectra was employed to further recognize the type of defects in photoreduced nano TiO_2_ samples. Figure 8a shows that modified RHT and R-P25 samples exhibit isotropic resonances ESR signal at g = 2.003, indicating the characteristic feature of oxygen vacancy trapped with one electron (Vo’s)^35,36^. While the absence of such signal in the ESR spectra of HT and commercial P25 TiO_2_ suggests that photoreduction treatment was responsible for the introduction of oxygen vacancies. The higher intensity of RHT indicates that the introduction of vacancies was easier in RHT than P25. ESR spectra also reveals the inexistence of Ti^3+^ species in photoreduced samples, as indicated by no response at g = 1.97^37,38^, which is consistent with the above XPS analysis.

We have succeeded in the synthesis of unique TiO_2_ hollow spiny nanocubes with high concentration of oxygen vacancies (Vo’s) and F doping. These structures of TiO_2_ nanocubes are respected to enhance the photocatalytic performance due to the improvement of both visible light utilization and separation of photogenerated charge carriers. In order to shed light on the photocatalytic activity, we firstly evaluated the photoelectrochemical (PEC) properties of the resulting materials.

Linear sweep voltammograms, transient photocurrent density as well as electrical impedance were recorded under the illumination of visible light (*λ* > 420 nm). The smallest hemicycle in the Nyquist plot of RHT, as showed in Fig. 8b, indicates that the RHT is more effective for charge separation and has lower resistance of charge transfer. As showed in Fig. 8c, RHT and R-P25 samples have higher photoactivity than its unreduced counterparts, HT and P25 samples. The same result can be also observed in Fig. 8d, during every periodic on-off cycle, all of the samples show a constant and sensitive response and the detected photocurrent density of RHT is ~3.0 times higher than that of HT. This enhancement can be attributed to the improved visible light response because of the increase of oxygen vacancies in photoreduced samples. Therefore, photoreduction treatment is a simple and efficient method to enhance the PEC property of nano TiO_2_ material. Compared with RHT, the crushed sample of DRHT shows relatively low photocurrent density, indicating that the well-defined hollow spiny nanocubes structure can facilitate the efficient charge separation and transportation. Besides, F^−^ doping is also an effective way to improve the PEC performance as shown in Figure S7, both RHT and RHT with NaOH solution treated samples have sensitive and robust response to visible light illumination, but the photocurrent density of RHT sample drop quickly after treating with NaOH solution, which is consistent with the above analysis.

**Figure 9:**
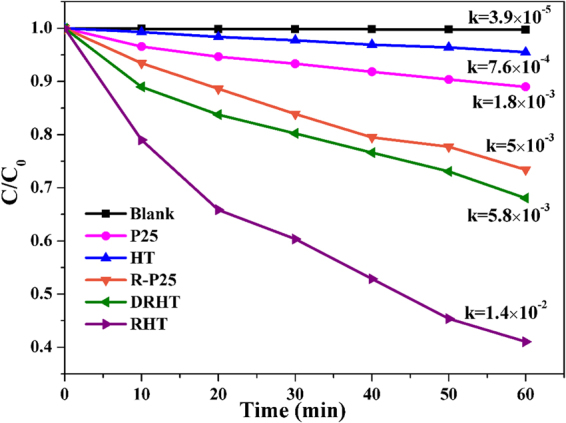
The photodegradation of phenol under visible light irradiation.

How to degrade aromatic compounds is of great interests due to their high chemical stability and high environment risk. We used phenol to testify the photocatalytic degradation activities of as-prepared TiO_2_ under visible light illumination. For the sake of contrast, a serious of blank tests with catalysts of commercial Degussa P25 and photoreduced R-P25, or even without catalyst, respectively, were performed (Fig. 9 and Figure S8). Normally, the self-degradation of phenol within the experiment period was negligible in the absence of any TiO_2_-based catalyst. And the photodegradation activities with pristine TiO_2_, such as P25 and HT, also appear to be quite slow. However, as for these modified TiO_2_ samples, the performance is significantly accelerated, indicating that photoreduction treatment indeed improve the photocatalytic degradation activity of TiO_2_ nanomaterials under visible light illumination. As expected, the highest apparent rate constant of RHT toward phenol photodegradation is 0.014 min^−1^, approximately 18 times higher than that of HT. This enhanced activity attributes to the extended response in visible light with relatively narrower bandgap and slower charge recombination. Furthermore, the hollow nanocubes with spiny structures and large surface areas considerably shorten the bulk diffusion length of photo-induced electron/hole pairs and contribute to more active sites, thus restraining bulk recombination. Dramatically, the activity of DRHT was decreased to 58.6% after the hollow spiny nanacubes was destroyed, although the surface area increases slightly from 191 to 211 m^2^/g. As mentioned above, the multiple reflections of light within the interior cavity of the unbroken allows for the improvement of light utilization efficiency and the corresponding catalytic activity. Furthermore, relatively high stable and recyclable property of RHT sample has been proved by the slight attenuation of efficiency toward phenol degradation (Figure S9b). The slightly decreased photocatalytic performance can be attributed to the catalyst loss during every centrifuging and drying process as well as the accumulation of organic matter on the surface of the photocatalyst.

## Discussion Section

Photocatalytic degradation of organic dyes is widely used as a model to evaluate the photocatalytic activity of TiO_2_. We have also used an organic dye, RhB, to evaluate the photocatalytic degradation activities of as-prepared TiO_2_ under visible light illumination. The photo-induced RhB decomposition reactivity under visible light illumination was displayed in Figure S9. As expect, the optimal degradation performance was achieved with RHT as catalyst, RhB was almost decomposed completely within 15 min. And R-P25 also displayed much better degradation ability when compared with P25. Therefore, it can be reasonably inferred that the relatively higher photodegradation efficacy mainly derived from oxygen vacancies induced by the moderate photoreduction treatment. To be mentioned, F^−^ doping also improves the photocatalytic activity for RhB decomposition, originating from the easier adsorption of the reactant molecules by the surface acidity enhancement.

Based on all the above analysis, defect-engineered F-doped TiO_2_ hollow spiny nanocubes was constructed with NH_4_TiOF_3_ mesocrystal as precursor, following by a facile photoreduction treatment. And the possible formation mechanism of TiO_2_ hollow structure from NH_4_TiOF_3_ is as follows^39^:

$$4{{\rm{NH}}}_{4}{{\rm{TiOF}}}_{3}+3{{\rm{H}}}_{3}{{\rm{BO}}}_{3}=4{{{\rm{NH}}}_{4}}^{+}+3{{{\rm{BF}}}_{4}}^{-}+{{\rm{OH}}}^{-}+4{{\rm{H}}}_{2}{\rm{O}}+4{{\rm{TiO}}}_{2}\,({\rm{anatase}})$$

During this process, under the environment of H_3_BO_3_ (aq), NH_4_^+^ and F^−^ were first dissolved from the surface of mesocrystal NH_4_TiOF_3_, resulting in the formation of {001} facet exposed anatase TiO_2_ nanothorny particles. Then, H_3_BO_3_ solution would enter into the interior zone of the precursor through the surface pores, achieving the gradual reactions between boric acid and NH_4_TiOF_3_ mesocrystal. At the same time, anatase TiO_2_ also crystallized to form the shell of nanothorns. After reacting for 4 hours, the interior of NH_4_TiOF_3_ completely exhausted and pure TiO_2_ hollow spiny nanocubes formed. The as-prepared TiO_2_ hollow spiny nanocubes as well as P25 would be further treated in photoreduction process. And the mechanism can be understood from Fig. 1. During this process, photo-induced holes and electrons were generated in TiO_2_ in hypoxia condition and UV-Vis light irradiation. The electrons were trapped by tetravalent titanium and reduce them to trivalent titanium. While holes were consumed by absorbed ethanol molecules to produce Ti-OH groups, and ethanol would be oxidized to acetaldehyde. Therefore, the temporally charge imbalance of TiO_2_ particles were formed, which would force O^2−^ to migrate to the surface and leave an oxygen vacancy^40,41^. Since a large number of F^−^ absorbed on the TiO_2_ surface, which were expected to diffuse into TiO_2_ lattice and occupy some of those oxygen vacancies^42^. However, in traditional doping process, it is much harder for fluorine ions to enter into TiO_2_ lattice, because their diffusion is easier in defective TiO_2−x_ than it in crystalline TiO_2_. So, there is also provide a facile strategy for doping. After the irradiation, O_2_ in air or dissolved oxygen in water would quickly oxidize those as-formed Ti^3+^. At the same time, the color of photoreduced TiO_2_ powders turned to earth yellow^43^.

In summary, the highly active hierarchical hollow spiny nanocubic TiO_2_ photocatalysts with F^−^ dopant and oxygen vacancies were successfully synthesized by the combination of topotactic transformation from NH_4_TiOF_3_ mesocrystal with photoreduction treatment. The introduction of more oxygen vacancies and F^−^ doping were achieved simultaneously during photoreduced process in virtue of the F^−^ release during structure transformation. The following factors contribute to the highest photocatalytic degradation efficiency of RHT sample: (i) the formation of defect energy levels and its resultant narrow band gap of TiO_2_ by the existence of Vo’s enhancing visible-light absorption ability; (ii) multi-reflections of the solar light caused by the hollow cubic spiny structure; (iii) the improvement of electrons and holes separation efficiency synergistically made by Vo’s, F^−^ codoping as well as the unique hollow architectures. Therefore, this feasible approach may open up new opportunities to design other photocatalysts for solar energy utilization and water purification in future.

## Methods Section

### Material

Commercial degussa P25 and Tetrabutyl titanate (TBOT, 98%) were obtained from Sigma-Aldrich Co. Ltd, boric acid, Isopropanol (99.5%) and ethyl alcohol (EtOH, 99.9%) were acquired from Sinopharm Chemical Reagent Co. Ltd, glacial acetic acid (HAc) as well as NH_4_F were obtained from Shanghai Macklin Co. Ltd. During the following experiment process, all of these chemicals and reagents were directly used without further purification or treatment.

### Preparation of NH_4_TiOF_3_ solid nanocubes

Typically, isopropanol (20 mL) and TBOT (4 mL) were blended in a 100 mL plastic beaker, then, stirring for 5 minutes to form solution A. The solution B, consisting of NH_4_ F (1.3065 g), deionized water (5 mL) and HAc (3 mL), was slowly added to the above solution A. In this process, the overall molar ratio of NH_4_F and TBOT was maintained at 3. Then the mixture was stirred vigorously for further 5 h under ice-bath and moved to 50 mL Teflon autoclave, keeping it in 180 °C for 12 h. After cooling quickly to room temperature, the white precipitate was washed and centrifuged with deionized water and ethanol, respectively. NH_4_TiOF_3_ was obtained after drying under vacuum for 10 h at 60 °C.

### Synthesis of Defect-engineered F-doped TiO_2_ Hollow Spiny Nanocubes

The as-prepared NH_4_TiOF_3_ solid nanocubes were placed in H_3_BO_3_ solution (50 mL, 0.5 M), keeping stirring for 5 minutes and recording as suspension C. The suspension C was maintained in 60 °C for 4 hours and dried after washing by water and ethanol. The obtained hollow TiO_2_ samples were donated as HT. F-doping reduced TiO_2_ with hollow spiny structure was derived from a facile photoassisted method, the acquired sample was labeled as RHT. The photoassisted process was performed under full spectrum illumination (250–750 nm) with a 300 W Xe lamp as light source (Beijing Perfectlight Technology Co. Ltd). In a typical procedure, HT sample or P25 (0.5 g) was dispersed in EtOH (30 mL). The suspension was put in a 50 mL quartz flask and stirred with 80 °C oil-bath, keeping bubbling by Argon with the irradiation of Xenon-lamp simultaneously, and an oxygen deficient atmosphere was created by liquid sealing other parts of flask. Maintaining this photoreduction process for 1 h, and then, directly dried for 2 hours at 150 °C. The above overall process was repeated for 3 times to gain RHT or R-P25. The hollow structure could be destroyed by ultrasound and grind and the corresponding sample was named DRHT. Defect-engineered TiO_2_ Hollow Spiny Nanocubes without F doping was achieved by mixing RHT sample with NaOH solution to remove fluorine.

### Material characterizations

A FEI Nova Nano SEM 450 field emission scanning electron microscope equipped with 18 kV accelerating voltage was used to take SEM images. TEM images, SAED and HRTEM images as well as the EDS mapping analysis were gained from JEOL JEM-2100F operating at 200 kV. XRD patterns were conducted on a SHIMADZU XRD-7000S diffractometer in the 2θ range of 10°–70° and the speed was 2°/min. XPS spectra test was carried out with ThermoFisher ESCALAB™ 250Xi. ESR spectra was recorded with Bruker A200-SRC at room temperature. JW-BK132F was used to measure BET specific surface areas at 77 K. Barrett-Joyner-Halenda (BJH) model was manipulated to calculate the pore diameter distributions and pore volumes. UV-Vis DRS spectra was carried out by a Perkin-Elmer Lamda 950 spectrophotometer in a region of 200–800 nm, BaSO_4_ was used as reference.

### PEC analysis

The photoelectrochemical (PEC) measurements were carried out with a standard three-electrode configuration on a CHI660E electrochemical workstation (Chenhua Instrument). Na_2_SO_4_ solution (0.2 M), Ag/AgCl electrode and Pt foil were used as the electrolyte, reference electrode and counter electrode, respectively. The working electrode was prepared by blending deionized water (0.4 mL), ethanol (0.1 mL), 3 mg photocatalysts and Nafion (20 *μ*L) together to form a homogeneous slurry. 0.2 mL of the resultant slurry was then dip-coated onto a 25 mm^2^ indium-tin oxide (ITO) glass, which was dried under 100 °C. Visible light was provided by a 300 W Xe lamp with 420 nm cut-off filter. Linear sweep voltammetry scans were performed under visible light illumination, scan rate was 20 mV/s and the potential range was −0.6 V to +1.0 V (versus Ag/AgCl). EIS were obtained in a frequency range of 1 to 10^6^ Hz. An operation voltage of 0.7 V (vs. Ag/AgCl) was imposed to record the transient response of photocurrent.

### Photocatalytic performance

The visible light photocatalytic performance of as prepared samples were testified by the degradation of RhB and phenol. Light source was a 300 W Xenon-lamp, and a 420 nm cut-off filter was adopted to filter out ultraviolet light. In a typical procedure, a 150 mL quartz beaker was used to contain TiO_2_ dispersion (60 mL, 0.5 g/L) and RhB or phenol solution (10 mg/L). The surface adsorption–desorption equilibrium was established by magnetic stirring for 60 min in the dark. During the whole experiment process, Xe lamp light source was put 15 cm above the suspension, 2 mL of the suspension was taken out from the reactor at 10 min interval. UV-vis spectrophotometer (Shanghai, UV-1800PC, AOE) was used to analyze the residual RhB concentration, and phenol concentration was monitored by Thermo Fisher UltiMate3000 HPLC. Apparent first-order rate constant *k* could help to characterize the photocatalytic degradation activity of phenol, which was calculated using the equation (1):

1 $$ln({C}_{0}/C)=kt\,or\,C={C}_{0}exp(-kt)$$

where *C* and *C*_0_ are the concentration of phenol after different irradiation time and the initial phenol solution concentration. The recycling experiments were executed for four consecutive cycles to test the reusability and stability of the photocatalysts by centrifugation after every cycle.

## Electronic supplementary material

Supplementary Information
