# Supplementary material for: Defect-engineered TiO2 Hollow Spiny Nanocubes for Phenol Degradation under Visible Light Irradiation
Source: Sci Rep. 2018 Apr 12;8:5904. doi: 10.1038/s41598-018-24353-8 (PMC5897375; doi:10.1038/s41598-018-24353-8)
Supplement: Supplementary file 1 — Supplementary Information [file 41598_2018_24353_MOESM1_ESM.docx]

**Supporting Information**

**Defect-engineered TiO_2_ Hollow Spiny Nanocubes for Phenol Degradation under Visible Light Irradiation**

Xiaolan Kang, Xue-Zhi Song, Ying Han, Junkai Cao, Zhenquan Tan*

School of Petroleum and Chemical Engineering, Dalian University of Technology, Panjin 124221, P. R. China.

*** Corresponding Author**

E-mail: tanzq@dlut.edu.cn (Z. Tan)


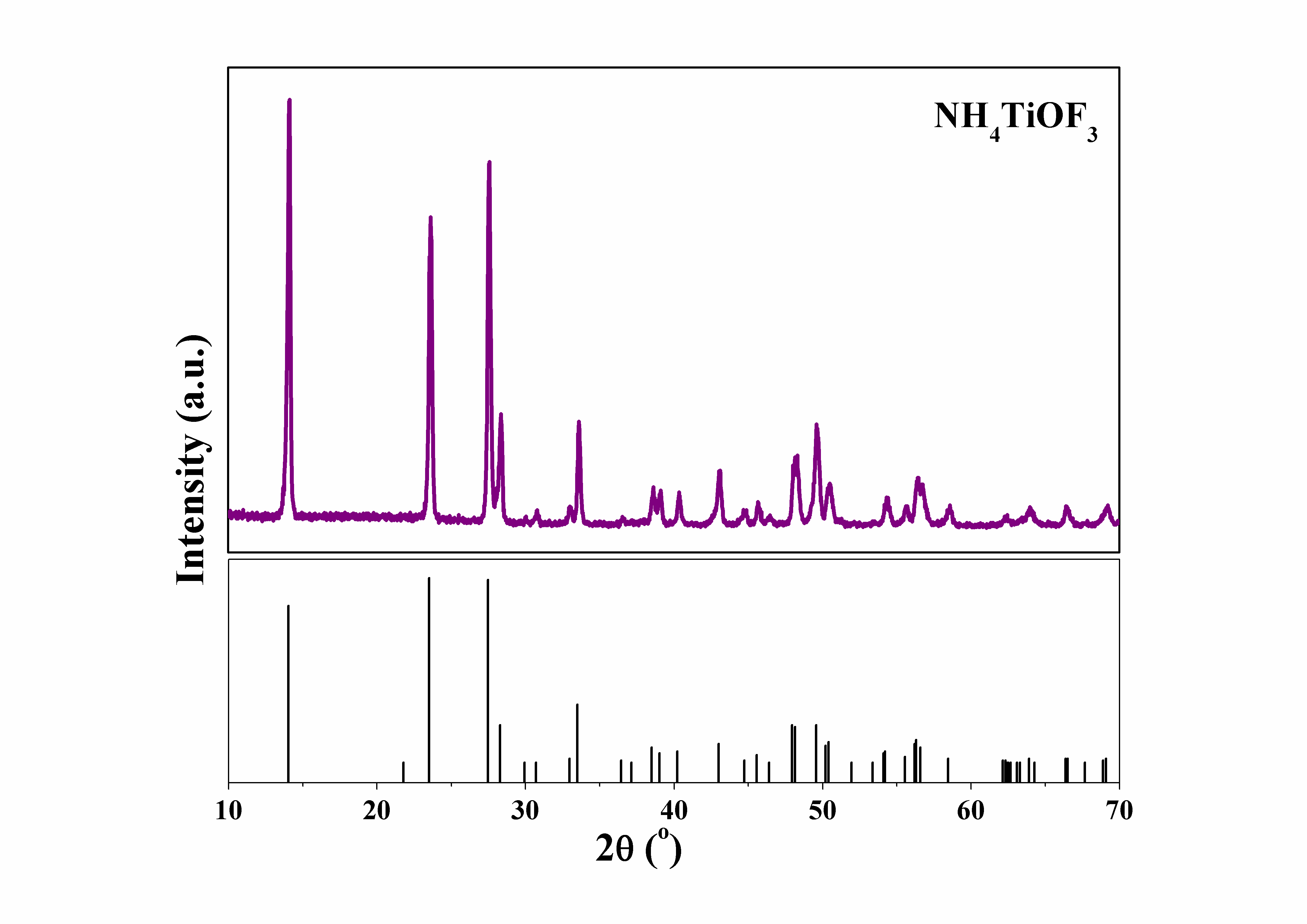


**Figure S1.** XRD patterns of NH_4_TiOF_3_.


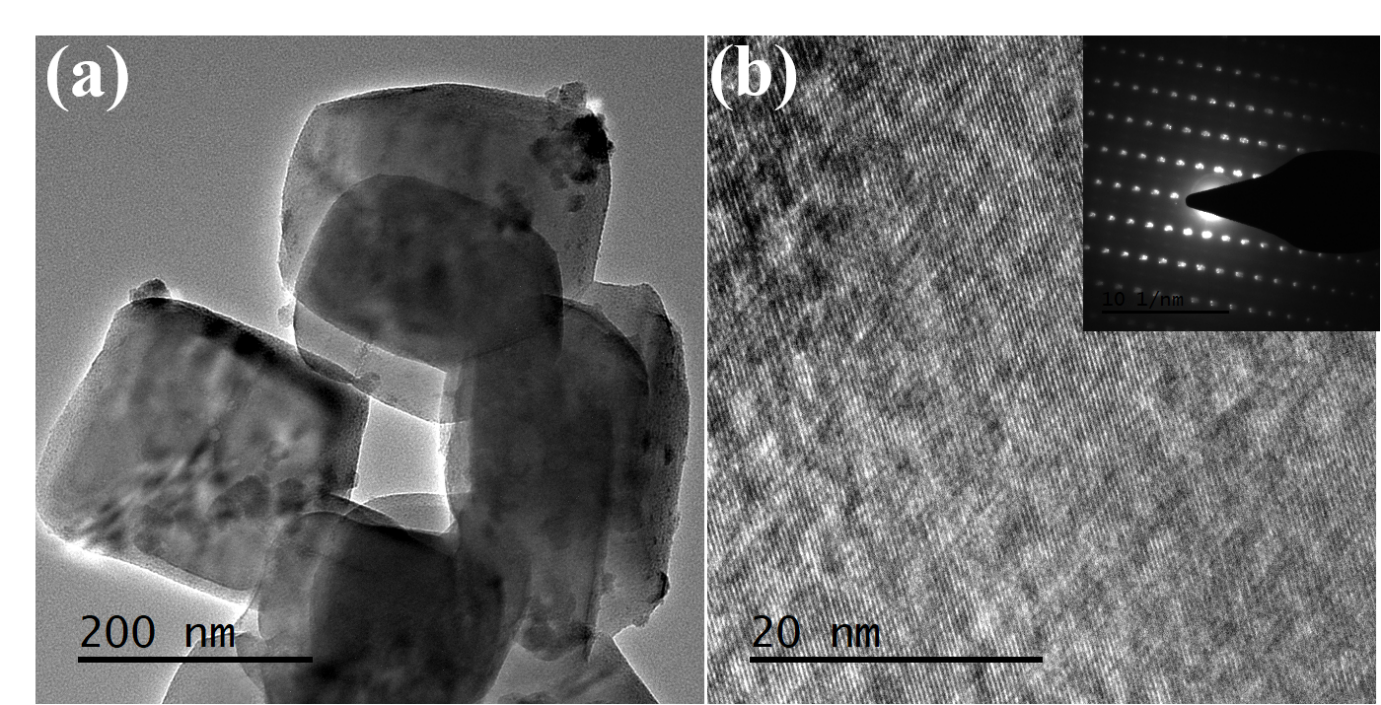


**Figure S2.** (a) TEM and (b) HRTEM images and SAED images (insert) of the as-prepared NH4TiOF3 sample.

**
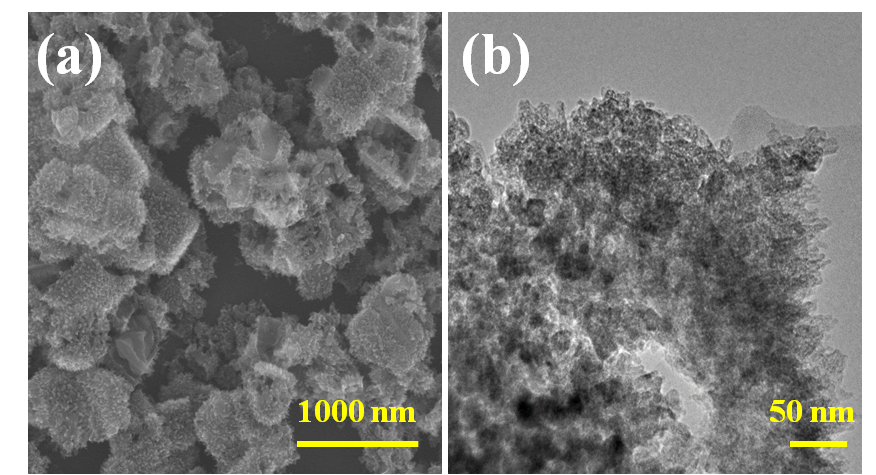
**

**Figure S3.** (a) SEM and (b) TEM images of DRHT.

**Table S1.** Structural characteristics of P25 and as-prepared samples.

| Sample | Surface area (m^2^ g^-1^) | Pore volume (cm^3^ g^-1^) | Pore size (nm) |
| --- | --- | --- | --- |
| NH_4_TiOF_3_ | 12.729 | 0.056 | 17.448 |
| RHT | 191.938 | 0.467 | 9.729 |
| DRHT | 211.376 | 0.604 | 11.425 |
| P25 | 53.695 | 0.227 | 16.939 |

**
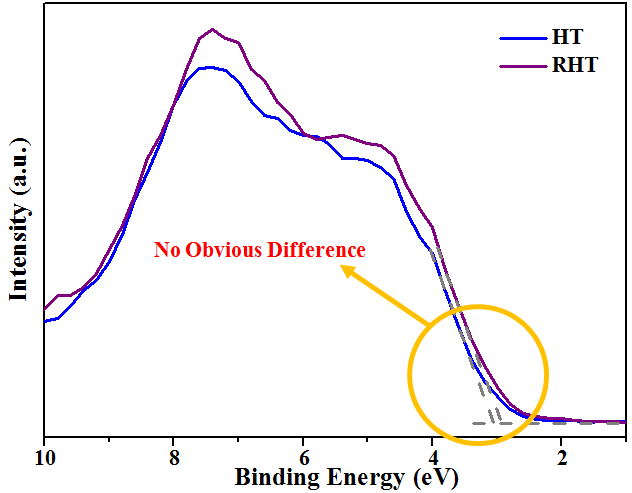
**

**Figure S4** Valence band of HT and RHT samples.


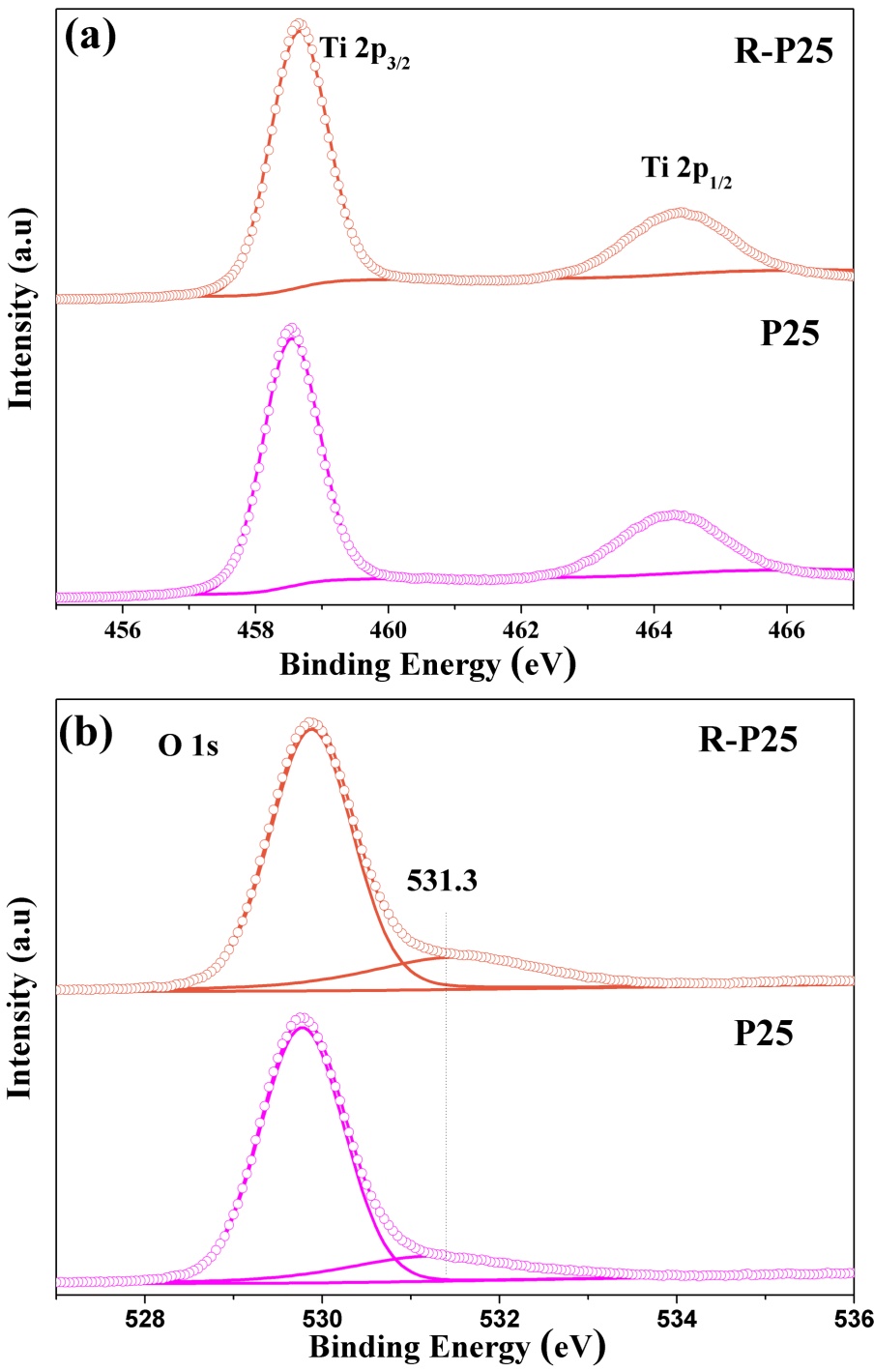


**Figure S5** XPS spectra of (c) Ti 2p, (d) O 1s for P25 and R-P25 samples.

**Table S2.** Ratios of different surface oxygen species of the as-prepared samples.

| Samples | Peak Area | | O_OH_/(O_OH_+O_L_) |
| --- | --- | --- | --- |
|  | O_OH_ | O_L_ |  |
| HT | 18779 | 47295 | 0.284 |
| RHT | 25947 | 78006 | 0.250 |
| P25 | 14632 | 61902 | 0.191 |
| R-P25 | 22769 | 73994 | 0.235 |


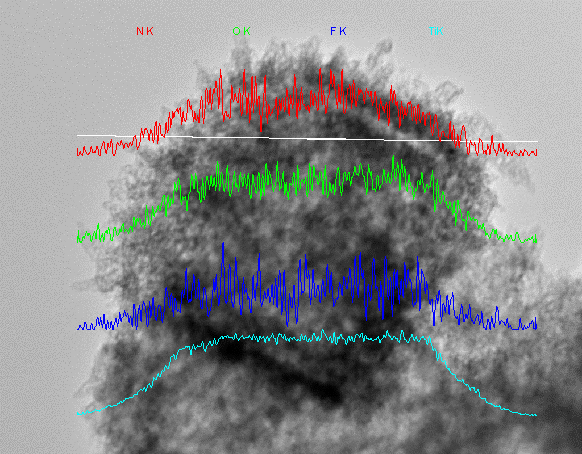

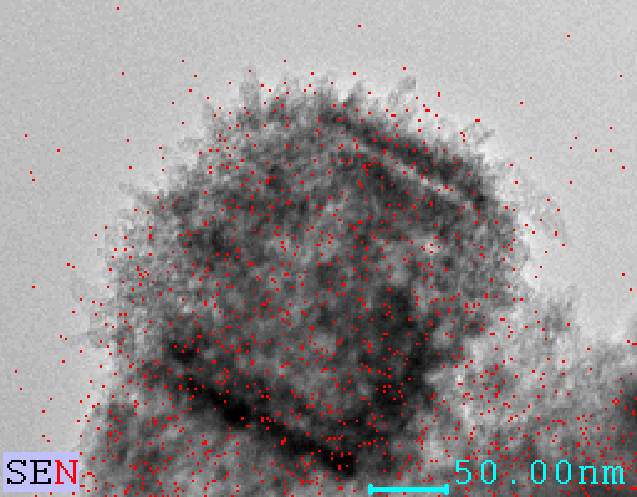

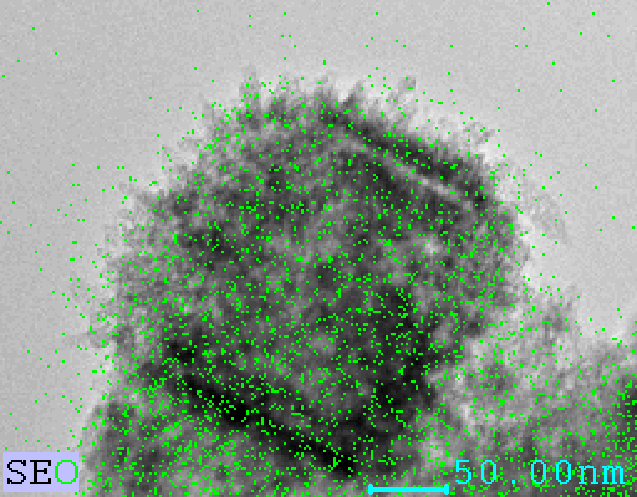

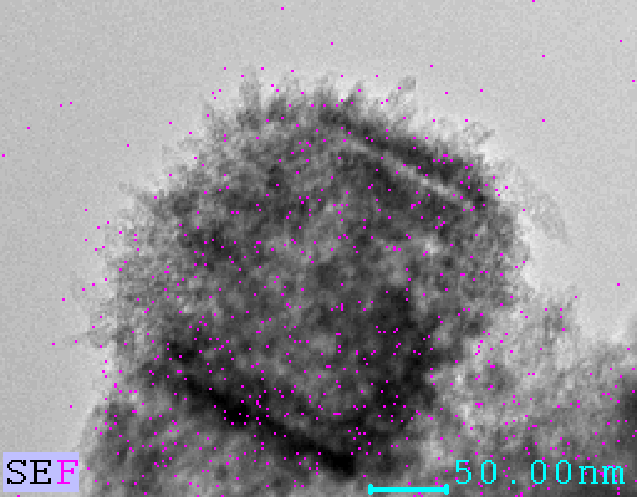

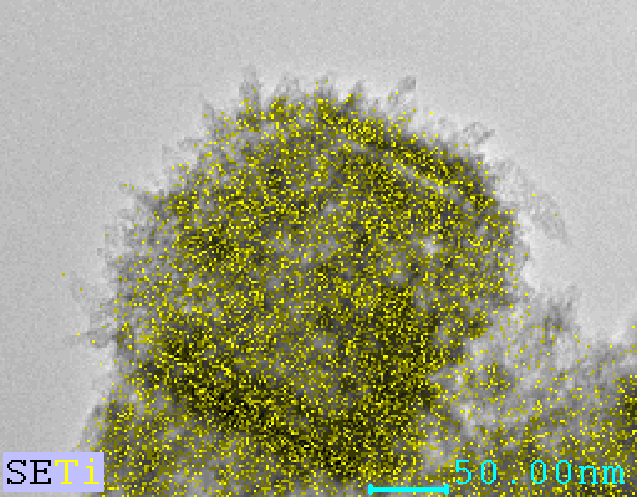

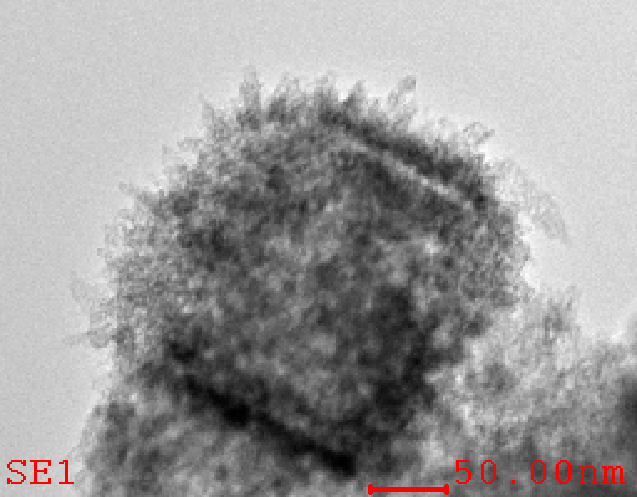


**RNT**

**(a)**

**(b)**

**(c)**

**(d)**

**(e)**

**(f)**

**Figure S6.** (a) STEM image of the sample of RHT, (b) STEM-EDS line scan and (c, d, e and f) STEM-EDS-mapping


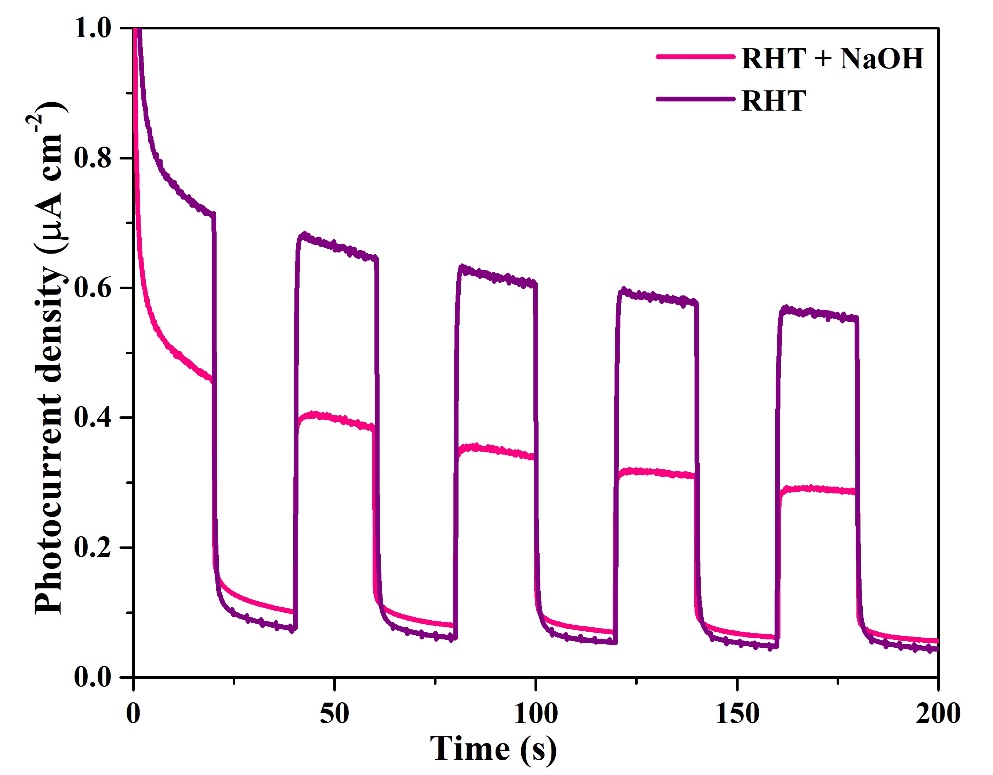


**Figure S7.** Linear sweep voltammograms under visible light irradiation of the as-prepared RHT and RHT with NaOH solution treated samples.


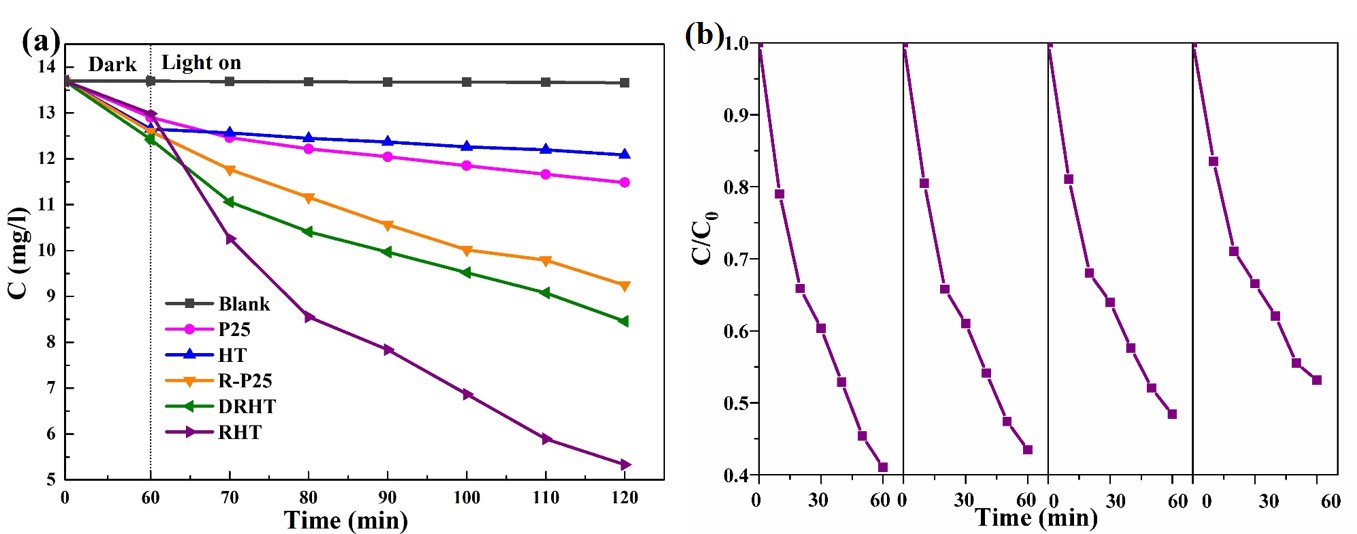


**Figure S8.** (a) The photodegradation of photodegradation of phenol with RHT under visible light irradiation, (b) and its corresponding Recycling tests.


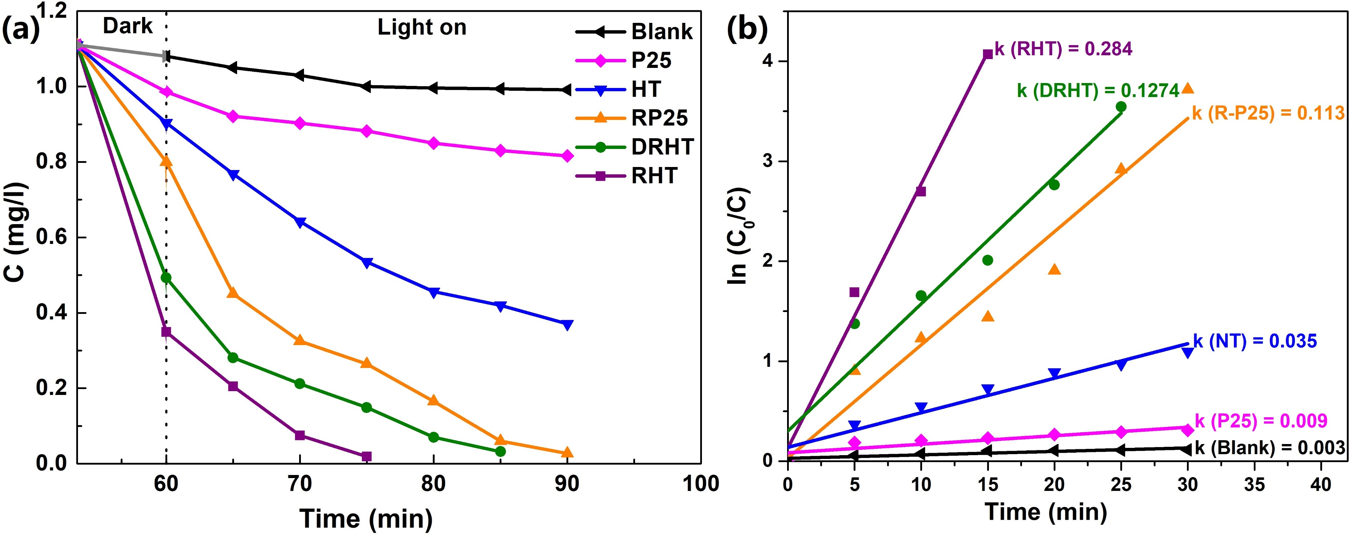


**Figure S9.** (a, b) the photodegradation of RhB under visible light irradiation.
